# The Potential Cardiotoxicity of Immune Checkpoint Inhibitors

**DOI:** 10.3390/jcm11030865

**Published:** 2022-02-07

**Authors:** Inbar Nardi Agmon, Osnat Itzhaki Ben Zadok, Ran Kornowski

**Affiliations:** 1 Department of Cardiology, Rabin Medical Center–Beilinson Hospital, Petach Tikva 4941492, Israel; osnat.itzhaki@gmail.com (O.I.B.Z.); ran.kornowski@gmail.com (R.K.); 2 Sackler School of Medicine, Tel-Aviv University, Tel-Aviv 6997801, Israel

**Keywords:** immune checkpoint inhibitors, cardiotoxicity, cardio-oncology

## Abstract

The use of immune checkpoint inhibitors (ICIs) as a mono- or adjuvant oncologic treatment is rapidly expanding to most fields of cancer. Alongside their efficacy, ICIs carry the risk of immune-related adverse events (irAEs) arising from misguided immune-mediated response to normal tissues. In the cardiovascular system, the cardiac toxicity of ICIs has been primarily related to the development of an acute, immune-mediated myocarditis; beyond this potentially fatal complication, evidence of an increased risk of cardiovascular events and accelerated atherosclerosis is emerging, as well as reports of other cardiovascular adverse events such as arrythmias, Takotsubo-like syndrome and vascular events. The absence of identified risk factors for cardiotoxic complications, specific monitoring strategies or diagnostic tests, pose challenges to the timely recognition and optimal management of such events. The rising numbers of patients being treated with ICIs make this potential cardiotoxic effect one of paramount importance for further investigation and understanding. This review will discuss the most recent data on different cardiotoxic effects of ICIs treatment.

Immune checkpoint inhibitors (ICIs) emerged in the last decade as a rapidly developing field of cancer treatments, and their use is expanding to a wide range of cancer fields [1]. In a simplified description, the ICIs re-activate cytotoxic T-cells, which were previously inactivated by the tumor, allowing them to recognize and target cancer cells. Currently used ICIs include antibodies against programmed death ligand-1 (PD-L1) or its receptor on T cells (PD-1), and against the immune regulatory protein cytotoxic T-lymphocyte-associated antigen 4 (CTLA-4) (Table 1) [2]. Alongside their efficacy, ICIs carry the risk of immune-related adverse events (irAEs) arising from misguided immune-mediated response to normal tissues. Approximately 60–80% of patients experience some irAEs under ICIs treatment, the most common being colitis, hepatitis, pneumonitis, hypophysitis and thyroiditis [3]. Up to a quarter of patients experience them at grade 3–4, as defined by the Common Terminology Criteria for Adverse Events (based on the severity of clinical manifestation and laboratory findings) [4]. The risk of irAEs, and their severity, increase when anti-CTLA4 and anti-PD1/PD-L1 are combined [5]. In the cardiovascular system, the cardiac toxicity of ICIs has been primarily related to the development of an acute, immune-mediated myocarditis, which is an uncommon but often has a fulminant course [6,7]. Beyond this potentially fatal complication, evidence of an increased risk of cardiovascular events and accelerated atherosclerosis is emerging, as well as reports of other cardiovascular adverse events such as arrythmias, Takotsubo-like syndrome and peripheral vascular events. The absence of identified risk factors for cardiotoxic complications or specific monitoring strategies or diagnostic tests, pose challenges to the timely recognition and optimal management of such events. The rising number of patients being treated with ICIs make this potential cardiotoxic effect one of paramount importance for further investigation and understanding. This review will discuss the most current data on different cardiotoxic effects of ICIs treatment. 

## 1. Autoimmune Myocarditis 

As is known, PD-L1 is expressed on myocytes, and its signaling path plays an important role in protecting the heart from autoimmune damage [8]. It was previously found that PD-1 gene-deficient mice developed dilated cardiomyopathy [9] and diffused myocarditis [10]. In 2016, Johnson et al. were the first one to publish two cases of fulminant and fatal myocarditis in patients treated with ICIs. Histological analysis confirmed myocardial infiltration of T-cell lymphocytes (both CD4+ and CD8+ T cells) and macrophages [11]. Increased reports of ICIs-related myocarditis have been published since, with estimated incidence ranging from 0.3% to greater than 1% [4,6,7,12]. Salem and colleagues used the World Health Organization’s (WHO) VigiBase pharmacovigilance database to retrieve reports of ICIs-associated cardiovascular (CV) events, of which 122 were reports of myocarditis, with fatality rate as high as 50% [3]. Data suggests that this immune-mediated myocarditis most commonly presents as an early manifestation, with a median time of 30 days after treatment initiation [6,7,13]; however, a wide variation exists, and some patients develop myocarditis later in the treatment course, even several months after starting ICIs treatment [7,14]. Patients who receive ICIs-combination therapy are at a highest risk of developing ICIs induced-myocarditis compared to a single-drug therapy. Although a pre-existing autoimmune disease and cardiovascular disease were suggested to increase the risk of ICIs-associated myocarditis, no other clear risk factors were identified [7,13,15,16,17]. Clinical presentation may vary from mild, non-specific symptoms, to fulminant course with cardiogenic shock and multi-organ failure [7,18]. Electrocardiogram (ECG) findings can range from normal ECG to tachycardia, ST-T changes, conduction abnormalities or arrythmias [13,19]. Laboratory examination shows elevated troponin, brain natriuretic peptide (BNP) or N-terminal (NT)-proBNP in most, but not all, patients. in previous cohorts, BNP was shown to be elevated in almost all patients, while troponin was elevated in less than half; this suggest that BNP should be part of routine evaluation [13,20]. In a substantial number of cases, a concomitant myositis exists, expressed in elevated creatine kinase [6,13,21,22]. Transthoracic echocardiography (TTE) is the first-line non-invasive examination to be performed when suspecting myocarditis. The echocardiographic findings may vary from a normal examination to reduced systolic and/or diastolic function, sometimes with concomitant pericardial effusion [7,12,13]. A recent study suggested a reduction in global longitudinal strain (GLS) on designated echocardiographic examination in an early sign of ICIs-induced myocarditis [23]. Cardiac Magnetic Resonance (CMR) is the gold- standard non-invasive modality in the diagnosis of myocarditis. CMR may demonstrate myocardial inflammation and necrosis in T1 and T2 sequences, and characteristic late gadolinium enhancement [4,23,24,25]. Endomyocardial biopsy is the gold-standard invasive test that provides a definite diagnosis. The pathologic picture resembles acute cellular rejection of the heart [13]; however, it is not often used due to its invasiveness and potential complications (Table 2). Since clinical presentation varies and there are no specific or pathognomonic imaging findings for the diagnosis of ICIs-related myocarditis, this serious and potentially fatal complication of ICIs treatment demands awareness and a high index of suspicion from the treating physician. ICIs-related myocarditis has been classified into four grades, based on the severity of symptoms, level of elevation of cardiac biomarkers, and echocardiographic findings; in most cases, patients require hospitalization for close monitoring and usually an intensive treatment, with a large portion requiring an intensive care unit setting [4]. Discontinuation of the ICIs and early initiation of high-dose intravenous glucocorticoids is the mainstay of treatment in cases of ICIs-related myocarditis; few case reports describe the addition of other immunosuppressive agents (e.g., tacrolimus, intravenous immunoglobulins, antithymocyte globulins) in severe cases [26,27]. Tailoring heart-failure treatment according to cardiac function and hemodynamic indices is also part of patient’s treatment. Importantly, the development of ICIs-related myocarditis also carries a significant therapeutic effect, as the permanent discontinuation of any treatment with an ICIs is advised for myocarditis grades 2–4 [4]. However, when no alternative oncologic treatment is available, a repeated trial of a different, single-agent, ICIs treatment may be carefully considered, under close cardiac monitoring [12,13].

## 2. Takotsubo-like Syndrome

Takotsubo syndrome (TTS) is an acute, mostly reversible, left ventricular (LV) systolic dysfunction, characterized by the classic echocardiographic findings of depressed LV functions along with akinetic apex (“apical ballooning”), in the absence of obstructive coronary artery disease. ICIs- associated TTS was reported in several studies and case-reports [4,13,28,29], including 13 cases which were reported in the WHO VigiBase pharmacovigilance database study [3]. Echocardiography is the cornerstone of noninvasive evaluation, but TTS is an exclusion diagnosis, which can be performed only after excluding acute coronary syndrome as the underlying cause (Table 2). Management of ICIs-related TTS is similar to that of ICIs-associated myocarditis, and includes the discontinuation of ICIs and considers the administration of high-dose corticosteroids, along with heart-failure medications and supportive care [17,27,29]. Most, but not all, TTS cases are reversible. When no alternative oncologic treatment is available, ICIs rechallenge may be considered after LV function has recovered, and is performed under close cardiac monitoring [28,29]. 

## 3. Pericardial Involvement 

Current data regarding ICIs- associated pericardial involvement are limited, but case-reports include pericarditis, pericardial effusion or tamponade [13,30,31]. The WHO VigiBase pharmacovigilance database study reported pericardial disease to be the second most common cardiac adverse event under ICIs treatment [3]. The median time for occurrence of pericardial disease was 30 days after the first ICIs treatment; furthermore, it was associated more with PD-1/PD-L1 therapy versus anti-CTLA-4, and was more common in patients with lung cancer compared with other cancers [3]. In a recent systematic review, 28 cases of ICIs-associated pericardial disease were identified, with the majority being life-threatening and severe [32]. It is important to remember that in many cases, pericardial effusion may represent the malignant involvement of the pericardium and not merely a cardiotoxic adverse effect of cancer treatment. Symptoms vary and may include dyspnea, chest pain, and hemodynamic instability due to large pericardial effusion which causes tamponade [33,34]. Physical examination may reveal typical pericardial chest pain and sometimes the presence of a friction rub on cardiac auscultation. ECG may range from normal to typical PR depression or diffused ST-T changes. Cardiac biomarkers should be measured, and, especially when elevated, the option of concomitant myocardial involvement should be considered (Table 2). ICIs treatment should be withheld. In stable cases, a conservative approach may by sufficient, with corticosteroids and sometimes colchicine and non-steroidal anti-inflammatory drugs (NSAIDs) as the mainstay of treatment [17]. In patients with hemodynamic instability, urgent pericardiocentesis may be indicated, and other cases of large pericardial effusion may benefit from surgical intervention with pericardial-window creation. These invasive options also provide the possibility of pathologic examination of pericardial fluid/pericardial biopsy in order to identify the underlying pathology [29,30,34].

## 4. Arrythmias 

Arrythmias, mostly supraventricular tachycardia but also atrial fibrillation, ventricular fibrillation and heart block, have been described in the context of ICIs therapy [4,13,28]. However, arrythmias can accompany myocarditis, pericardial involvement, hyperthyroidism, electrolyte disturbances and many other clinical scenarios; therefore they are mostly considered as a secondary manifestation and not a direct effect of the ICIs treatment itself [28].

## 5. Accelerated Atherosclerosis and Increased Risk of Cardiovascular Events

Immune checkpoints are established negative regulators of atherosclerosis- and arterial wall disease, which involves a lipid-driven chronic inflammatory process, in which T cells play a dominant role. For example, mice lacking PD-1/PD-L1 pathways demonstrated an increase in atherosclerotic plaque [35], while CTLA4 overexpression in hyperlipidemic mice resulted in an athero-protective profile [36]. Under ICIs treatment, the atherosclerotic plaque was shown to display an activated T-cell profile, which not only promotes the progression of atherosclerotic lesion formation, but also drives the process towards vulnerable plaques that may trigger myocardial infarction or ischemic stroke due to plaque rupture [37,38,39].

Recent data suggest an increased incidence of myocardial infarction in ICIs trials [17]. Drobni et al. reported a 4-fold increase in a composite CV outcome in cancer patients treated with ICIs compared with cancer patients who do not receive such treatment [39]. Interestingly, while the imaging sub-study found that a concomitant use of statin was associated with a reduced progression rate of the atherosclerotic plaque compared with cancer patients receiving ICIs but no statin, in the matched-control and cross-over cohorts described above, no clear association was found between the “classic” CV risk factors (e.g., diabetes, smoking, previous history of ischemic heart disease, etc.) and the elevated CV risk accompanying ICIs treatment [39]. The factors putting certain patients at higher risk for CV events under ICIs treatment and the most appropriate strategy for monitoring and treating such patients are yet to be determined, and deserve further studies. When acute coronary syndrome is suspected in a patient under ICIs treatment, evaluation and treatment should follow common guidelines (Table 2) [40,41]. In cases in which coronary angiography does not indicate an atherosclerotic etiology for the event, coronary vasculitis should be taken into consideration, as discussed below. 

## 6. Vasculitis

Vasculitis disorders observed in association with ICIs can affect vessels of any size, but were most commonly reported in larger vessels, particularly temporal arteritis [30]. An immune-mediated inflammation of the arterial walls is thought to be the leading pathogenesis. The main concern with temporal arteritis is the risk of permanent blindness with ophthalmic involvement; therefore, a high index of suspicion is required for the early detection and initiation of appropriate immunosuppression treatment, which relies mainly on high-dose corticosteroids [3,30].

## 7. Clinical Implications and Conclusions

**Figure 1:**
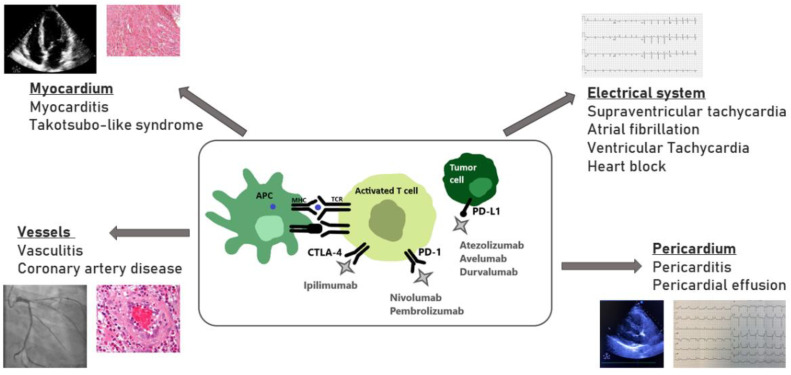
Potential cardiotoxicity of the immune checkpoint inhibitors. APC- antigen presenting cell; CTLA-4- cytotoxic T-lymphocyte-associated protein 4; MHC- major histocompatibility complex; PD-1- programmed cell death protein 1; PD-L1- programmed death ligand 1; TCR- T cell receptor.

Myocarditis is a well-established life-threatening cardiotoxic adverse event of ICIs therapy. However, with the relative novelty of the ICIs, together with their rapid expansion into all fields of oncologic treatments, short and long-term complications that have not yet been revealed are highly likely (Figure 1). Moreover, most clinical trials have excluded elderly patients and those with a history of CV disease, making it likely that the effects of ICIs on atherosclerosis have been underestimated so far. The increased reporting over time of ICIs CV adverse events represents both the expansion of ICIs treatment, as well as the increased awareness of healthcare professionals to these potential events. The identification of patients at higher risk for CV adverse events is of high importance, as this will allow for a closer monitoring and surveillance, and possible earlier interventions using multidisciplinary cardio-oncology collaborative units. 

The European Society of Cardiology (ESC) recently published a position statement which recommends a baseline cardiac assessment of all patients scheduled for ICIs treatment initiation, including a clinical history and risk factor assessment, ECG, cardiac troponin, BNP or NT-proBNP and echocardiogram. Having these parameters available at baseline will allow for a better evaluation of patients later on, in case a cardiotoxic adverse event is suspected. In patients categorized as high-risk patients, the current recommended surveillance during ICIs treatment includes ECG, cardiac troponin and BNP/NT-proBNP assessment before ICIs doses 2, 3 and 4; if normal, then further assessment can be reduced. In case of new troponin or BNP elevation, ECG or echocardiographic abnormality, the patient should be referred to a cardio-oncology specialist. Any patient with a new cardiac symptom should undergo prompt evaluation including ECG, echocardiography, cardiac troponin and BNP/NT-proBNP and should be referred to a cardio-oncology specialist if any new abnormalities arise [21].

Given the increasing number of patients treated with ICIs, further research is needed to establish standards of monitoring and best practical management of patients in all potential manifestations of ICIs cardiotoxicities. Once ICIs-related cardiotoxicity is developed, a close collaboration is needed in a multi-disciplinary team in order to outline the most appropriate management and best treatment options in both a cardiac and oncologic sense.

## Figures and Tables

**Table 1:** ICIs currently approved by the United States Food and Drug Administration (FDA) (in chronologic order of approval), with selected common FDA approved indications (mostly given in metastatic/unresectable disease, and in some cancers as an adjuvant therapy for earlier stages).

Drug Name	Molecular Target	Common Indications by FDA Approval
Ipilimumab	CTLA-4	Melanoma, NSCLC, hepatocellular carcinoma, renal cell carcinoma, malignant pleural mesothelioma
Nivolumab	PD-1	Melanoma, NSCLC, colorectal cancer, esophageal cancer, gastric cancer, hepatocellular carcinoma, renal cell carcinoma, Hodgkin’s lymphoma, Urothelial carcinoma
Pembrolizumab	PD-1	NSCLC, triple negative breast cancer, cervical cancer, cutaneous SCC, esophageal cancer, gastric cancer, head and neck SCC, hepatocellular carcinoma, melanoma, Merkel cell carcinoma, primary mediastinal large B-cell lymphoma, renal cell carcinoma, urothelial carcinoma
Atezolizumab	PD-L1	hepatocellular carcinoma, melanoma, NSCLC, urothelial carcinoma
Avelumab	PD-L1	Merkel cell carcinoma, renal cell carcinoma, urothelial carcinoma
Durvalumab	PD-L1	NSCLC, small cell lung cancer

CTLA-4- cytotoxic T-lymphocyte-associated protein 4; NSCLC- non small cell lung cancer; PD-1- programmed cell death protein 1; PD-L1- programmed death ligand 1; SCC- squamous cell carcinoma.

**Table 2:** Different diagnostic modalities for main actue ICIs-cardiotoxicities.

	ECG	Circulating Biomarkers	Echocardiography	CMR	Other
Autoimmune- mediated myocarditis	Can range from normal ECG to tachycardia, ST-T changes, conduction abnormalities or arrythmias.	Troponin and BNP are usually elevated, but may also be normal. CPK may be elevated with concomitant myositis.	Findings may range from normal function to reduced systolic and/or diastolic function. Reduction in GLS may be an early marker to myocardial injury. Pericardial effusion may be present.	May demonstrate myocardial inflammation and necrosis in T1 and T2 sequences, with characteristic late gadolinium enhancement.	Endomyocardial biopsy will show predominant lymphocytic infiltration.
Takotsubo-like syndrome	May mimic acute coronary syndrome, with ischemic chages	BNP elevation may be significantly higher than troponin elevation.	Acute LV systolic dysfunction. Classically apical akinesia (“apical ballooning”)	Left ventricular impairment without evidence of active myocarditis.	Diagnosis can be done only after excluding acute coronary syndrome.
Pericardial involvement	May range from normal to typical PR depression or diffused ST-T changes.	When troponin is elevated, concomitant myocardial involvement should be suspected.	May demonstrate pericardial effusion.	May demonstrate active pericardial inflammation.	
Myocardial infarction	New ischemic changes (sg, ST elevation/ depression or T-wave inversion)	Troponin elevation.	Usually, new regional-wall motion abnormality will be present.	May demonstrate regional-wall motion abnormality and characteristic mid-wall late gadolinium enhancement.	Coronary angiography for invasive diagnosis.

BNP- brain natriuretic peptide; CMR- cardiac magnetic resonance; CPK- creatinine phosphokinase; ECG-electrocardiogram; GLS- global longitudinal strain; ICIs-immune checkpoint inhibitors; LV- left ventricle.

## Data Availability

Not applicable.
